# The Study of Etiology; Its Importance and the Needed Methods to Insure Success

**Published:** 1876-08

**Authors:** N. S. Davis

**Affiliations:** Chicago, Ill.


### ON THE STUDY OF ETIOLOGY; ITS IMPORTANCE, AND THE NEEDED METHODS TO INSURE SUCCESS.

By N. S. DAVIS, A. M., M. D., Chicago, Ill.

(Read to the Chicago Medical Society, May 1st, 1876.)

To the thoughtful practitioner there is no department of medical science in so unsatisfactory a condition as that which relates to the *causes* of disease. With a steadily increasing tendency in the professional mind to refer all acute diseases, whether epidemic, endemic or sporadic, to specific causes or viruses, capable of propagation by fermentative or zymotic action, there is an equal tendency to indulge in hypotheses instead of carefully observed facts. It is so much easier, and takes so much less time, to invent a plausible theory and then use it as though it had been already verified, than to patiently observe a sufficient number and variety of facts to afford a reliable deduction, that few can resist the temptation to follow the former course. It is amusing sometimes to notice how readily, and apparently unconsciously, we assume one purely hypothetical proposition and then make it the basis of a lengthy paper or a monograph. For instance, in a paper read to another society in this city a few months since, the writer assumed substantially, first, that every *specific* disease must have a specific cause; second, that cholera infantum and dysentery were *specific* diseases, hence, logically, they must each depend on a specific infectious cause, and should be put together in one group; while the cases of simple diarrhœa, not being *specific*, and consequently not dependent on a specific infection, should constitute a second group. This latter group he again divides into no less than *five* classes or varieties, according to their assumed *causes*, namely: 1st, from the reflex irritation of dentition; 2d, from "sepsis or toxæmia;" 3d, from "acute indigestion;" 4th, from malarial infection; 5th, from intestinal catarrh, produced by cold and damp. Thus, in a paper written avowedly for the purpose of separating the different bowel affections of children from each other, we have one group embracing dysentery and cholera infantum, and another embracing five varieties of "simple diarrhœa," all based on supposed differences in their *causation*. There is no intimation as to how we are to distinguish one of these varieties from another at the bedside of the patients. To sustain the idea that dysentery is a *specific disease* produced by a specific cause, it is declared to be something more than, and different from *ileo-colitis*, although presenting similar symptoms and the same post-mortem appearances. He speaks of the "germs of cholera" bringing forth cholera "as immutably as the grain of corn brings forth corn, and no other grain," with as much confidence as though such *germs* had been fully identified and their specific influence established. And yet he says, "whether its (cholera infantum) essential pathology lies in the presence of bacteria or a blood ferment, we cannot perhaps declare to-day"; and then, on the same page, makes the following declaration: "Respecting the real causation of cholera infantum we must confess our utter ignorance, for nothing is positively known of it." Mark the progress: First, specific *germs* as the cause of a disease are not only spoken of confidently, but an *immutable law* of action assigned them. Second, we are reminded that it is *uncertain* whether the essential pathology of the same disease lies in the presence of bacteria or a blood ferment. Third, he confesses "utter ignorance" of the real causation of the disease, saying that "nothing is positively known of it."* To make confusion more confounding, it will be noticed that in the second proposition just stated, the "essential pathology" of disease and the *cause* are made identical. In other words, the *cause* and the *thing caused* are one and the same. I have not alluded to this essay of one of our most intelligent physicians for the purpose of criticising it, or of finding fault with its author, but simply to illustrate the point under consideration. The same disposition to assume the existence of causes acknowledged not to have been verified by any adequate degree of proof, is manifested by writers of the highest authority.

Thus, in Ziemssen's Cyclopœdia of the Practice of Medicine, now in the process of publication, etiology enters largely into the basis of classification or arrangement of diseases. And a large portion of the most important diseases that come under our observation are designated acute and chronic "infectious diseases;" thereby implying that they depend for their essential cause upon some specific infectious poison. In the introductory chapter to the first volume, Liebermeister says: "Under the name of *Infectious Diseases* we grouped together those affections which we know, or at least *believe*, must originate through the infection of the system with certain peculiar poisonous matters, and which are mainly distinguished from the ordinary poisons by the fact that they can reproduce themselves, under favoring conditions, to an endless degree." Again

* See Chicago MEDICAL JOURNAL AND EXAMINER, Jan., 1876, pp. 16 and 17.

he says, "the poisons of infectious diseases can reproduce themselves, and to an unlimited extent." Although this eminent writer, in this same introductory chapter, freely admits that it is still an open question whether the supposed *infectious poisons* are living germs —"contagium vivum"— or inorganic materials; and that "in the large majority of infectious diseases the poisons by which they are called into activity have been hitherto unknown;" thereby clearly admitting the whole to be nothing more than a plausable hypothesis; yet the simple fact that a long list of important diseases are classed under that name will lead others to speak and write volumes in a style of positive phraseology, as though every hypothetical infectious poison were a demonstrated fact. Indeed, Liebermeister himself, while fully admitting that his doctrine of a "contagium vivum," or living self-propagable germs as causes of disease, was first rendered popular by the investigations of Lieuenhæch, and subsequently abandoned, and now again revived only under more favorable auspices, but requiring for its establishment much additional and careful investigation, is constantly using expressions which fairly imply that such *germs* and their action as causes of disease are already demonstrated facts. It is this almost universal disposition to make a limited number of imperfectly observed facts the basis of plausible hypotheses, or to boldly assume the existence of germs, miasms, contagions, etc., as causes of disease, and to endow them with just such properties and capabilities as are necessary to make them serve our purposes, that has filled the pages of medical literature with a heterogeneous mixture of facts and fancies, or assumptions, sufficient to exhaust the patience of a Job and confound the wisdom of a Solomon. Yet, when we remember that much of our progress in the rational treatment of disease, and nearly all of advancement in sanitary science, must depend on the extent and accuracy of our knowledge of *etiology*, we shall realize the importance of any measures really calculated to bring into existence well devised plans for systematic observation of facts until they afford reliable deductions, instead of fragmentary observation sufficient only for the suggestion of plausible theories.

The difficulties encountered in the study of this branch of medical science are more numerous and troublesome than in any other department. This arises from the fact that the questions presented for solution are more complicated. In other words, the factors or elements necessary to be taken into consideration in solving every problem concerning the causation of disease, are not only numerous, but many of the factors are themselves complex. As living beings we move in an atmosphere containing many and constantly varying elements and properties, yet holding the most intimate relations to the vital processes within us; and we are subject to the impress of forces, material and mental, of the most varied character. In studying pathology we have directly before us the phenomena during life, and the results in the diseased structures after death. In the study of therapeutics we hold the medicinal agents in our own hands, control their administration and have the opportunity to watch their effects. But in the study of etiology the agents and forces active in the production of disease, neither warn us of their approach nor tarry for our inspection. Yet, with the exception of the specific viruses of variola, rubeola, scarlatina, syphilis, and a few others, there is no doubt but that all the causes of disease, whether consisting of living germs — *contagium vivum*,— inorganic poisons, or simple modifications of the natural elements and forces that surround us, act only under certain laws or "favoring conditions." Hence the first steps of real permanent progress must consist in determining these favoring conditions. For, though Liebermeister and others often broadly assert that it is the peculiar characteristic of infectious poisons that they are capable of self-propagation, and that to an unlimited extent, whether in the body or exterior to it, yet this is true *only* in the presence of certain necessary conditions. That the prevalence of many important diseases is influenced to a very great degree by season of the year and local sanitary conditions, is apparent to all. For instance, the bowel affections of children, cholera, periodical fevers, yellow fever, etc., prevail almost wholly in the summer and autumn, while pneumonia, croup, catarrh, and rheumatism prevail chiefly in the winter and spring. But if we attempt to go beyond these general and easily recognized facts, and inquire for the particular element or elements more immediately concerned in the production of any one of these diseases, we are met by difficulties not easily overcome.

Chief among these difficulties, is the absence of continuous carefully recorded facts, both in regard to the appreciable conditions and qualities of the atmosphere, and the *date* of the initial symptoms of diseases. We have an abundance of meteorological tables presenting the *mean* daily, monthly, and annual temperature, the moisture, the rainfall, and the extremes of heat and cold; but the equally important items, relating to the direction of the winds, the daily variations of temperature, and the electric and ozonic conditions of the atmosphere, are either omitted or so imperfect as to be of little value. On the other hand, recorded observations in regard to the date of the commencement of the active phenomena of disease, are almost entirely absent from the pages of our medical literature. The statistics of mortality give us only the date of deaths, but neither inform us correctly concerning the whole number of cases of sickness, nor the date at which any of the cases begin. To study satisfactorily the causes of all acute diseases not known to be propagated by specific contagion engendered in the body of the sick, we need three distinct series of observations and record, made simultaneously and in a sufficient number of places, and continued through a series of years.

One of these should consist of a complete registry of atmospheric conditions, including electricity and ozone. The observations and records now made under the direction of the signal service bureau of the general government, include a sufficient number of stations, and are sufficiently complete, except in regard to electricity and ozone. Another series of observations should be made in the same localities where the meterological records are kept (so far as these are inhabited) by active and intelligent practitioners of medicine, who should endeavor to ascertain and record the exact date of the commencement of active symptoms in all attacks of acute disease coming under their observation, and the date of relapses when such occur. A third series should consist in a systematic microscopical examination of the atmosphere, the dew and the drinking water, in regard to a uniform plan, and in as many of the same localities where the meteorological and clinical observations are made, as competent observers could be found who would faithfully carry on the work. It is obvious that a careful comparison of the results of the first two series of observations would enable us to know the exact relation between given conditions of the atmosphere and the development of different forms of disease. It would enable us further to trace the relation between the *degree* of certain atmospheric conditions and the *severity* of the attacks of disease. While the third series of observations, compared with the results of the first and second, would go far toward deciding the question whether the coincidence of certain atmospheric conditions developed special organic germs; and whether these bore any direct relation to the commencement, progress and decline of certain forms of disease.

It is only by carefully considered methods of investigation, established in many localities, and executed faithfully through a series of years, that all the data can be obtained for determining the true etiology of acute diseases, whether endemic, epidemic or sporadic. With the aid now attainable from the signal service bureau of the general government, covering the most expensive part of the series of investigations needed, this and all similar medical societies could easily plan and execute such arrangements as would secure the coincident clinical and microscopical observations and records. Let as many of the members engaged in active general practice as possible, make it a rule to ascertain and note in a memorandum book for that purpose every case of acute disease coming under their observation, specifying as exactly as possible the *date* of the commencement of symptoms of disease. Let them once in three or six months tabulate these. Let a sufficient number be engaged to make daily examinations with the microscope.

Let another set keep a daily record of the electric and ozonic conditions of the atmosphere. Let the tabulated statements of all of these be reported to a committee appointed for that purpose, once a year. And when this committee has carefully compared the several series of observations and the meterological records, let them condense the same into one tabulated report, putting one copy on file and sending one to the committee on the same subject appointed by the proper section of the American Medical Association; by whom it should be carefully compared with similar reports from all other societies, and the results published annually in the transactions of the Association.

It is true that such a scheme would require, on the part of all engaged in it, much patience and pains-taking labor; and this, above everything else, is just what the science and literature of our profession most need. At present we are overwhelmed in every direction by detached facts, partial or incomplete observations, imperfect records, and hasty generalizations. And if this, and kindred societies, acting in concert with the proper sections of the American Medical Association, would give more time to the devising of more complete methods of investigation, and the patient supervision of their execution until completed, they would do more to advance the science and art of medicine in five years, than could be accomplished in twenty by receiving and discussing reports and papers presented by individual members in the ordinary way. And not only this, but every member engaging in such systematic observations, and the keeping of records, would be doubly compensated by the increased mental discipline, habits of observation, and accuracy of knowledge which he would acquire.

